# Silver nanoparticles promote procoagulant activity of red blood cells: a potential risk of thrombosis in susceptible population

**DOI:** 10.1186/s12989-019-0292-6

**Published:** 2019-02-14

**Authors:** Yiying Bian, Keunyoung Kim, Thien Ngo, Inho Kim, Ok-Nam Bae, Kyung-Min Lim, Jin-Ho Chung

**Affiliations:** 1 College of Pharmacy, Seoul National University, Seoul, 151-742 South Korea; 2 Seoul National University Hospital, Seoul, 03080 South Korea; 3 College of Pharmacy, Hanyang University, Ansan, Gyeonggido 426-791 South Korea; 4 College of Pharmacy, Ewha Womans University, Seoul, 120-750 South Korea

**Keywords:** Silver nanoparticles (AgNP), Toxicity, Thrombosis, Red blood cells (RBCs), Cancer

## Abstract

**Background:**

Silver nanoparticles (AgNP) are widely used in medical practices owing to their distinct antibacterial, antiviral and anticancer activities. However, with increasing use of AgNP, concerns over its potential toxicity are also escalating. Here, we demonstrated the potential thrombotic effect of AgNP which was mediated by the procoagulant activity of red blood cells (RBCs).

**Results:**

In freshly isolated human RBCs, AgNP, but not silver microparticles (AgMP), elicited morphological changes, phosphatidylserine (PS) exposure and microvesicles (MV) generation, the key indicators of procoagulant activity in RBCs at concentration ranges (≤ 100 μg/mL) that were free of significant hemolysis. In line with this, AgNP potentiated thrombin generation and adherence of RBCs to endothelial cells, while AgMP did not. Oxidative stress, intracellular calcium increase and ATP depletion were found to underlie the procoagulant effects of AgNP, which led to altered activity of membrane aminophospholipid translocases. These in vitro findings were well reproduced in rat in vivo, where intravenously exposure to AgNP promoted venous thrombosis significantly. Of note, RBCs isolated from cancer patients, who inherently convey the risk of thrombogenesis, were more sensitive to the procoagulant effects of AgNP. In addition, AgNP significantly potentiated the procoagulant effects of a chemotherapeutic drug, paclitaxel.

**Conclusion:**

Collectively, these results suggest that AgNP may have prothrombotic risks by promoting procoagulant activity of RBCs and caution shall be taken for its use in the population sensitive to thrombosis like cancer patients.

**Electronic supplementary material:**

The online version of this article (10.1186/s12989-019-0292-6) contains supplementary material, which is available to authorized users.

## Background

Silver nanoparticles (AgNP) are widely used for therapeutic interventions and diagnosis in medical practices and have gained increasing popularity as drug carriers, nanoprobes, bio-imaging and labeling agents [1, 2]. Along with increasing application of AgNP in nanomedicine, however, concerns are also escalating over its potential toxicity against human health [3–5]. Potential adverse effects of AgNP against human health have been first illuminated at a concentration range of 25 ~ 500 μg/mL for 10 ~ 72 h duration *in vitro*, with respect to its cytotoxicity on human cell lines, reactive oxygen species (ROS) generation, oxidative stress, cell-cycle arrest, pro-apoptotic effects and genotoxicity [5–7].

AgNP readily enters the systemic circulation [8–10]. Accordingly, much attention has been paid to the potential cardiovascular toxicity of AgNP due to an easy access of AgNP to the tissues of cardiovascular system like blood cells, heart, and blood vessels [11–13]. Previously, we demonstrated that AgNP can activate platelets, which ultimately contributed to increased thrombosis [12]. Thrombosis and embolism are serious life-threatening complications of various diseases with hypercoagulable states that include cancer [14], nursing home confinement, surgery, trauma [15], and heavy metal intoxication [16, 17]. In addition to platelets, red blood cells (RBCs), a major cellular component of blood, can participate in venous thrombosis through facilitating coagulation cascade and clot formation by providing a site for the assembly of prothrombinase and tenase complexes [17]. Externalization of phosphatidylserine (PS), an anionic phospholipid, to the outer leaflet of lipid bilayer is key in this process, which is primed through intracellular calcium increase, ATP- and thiol-depletion [18]. Oxidative stress has been well-established to amplify PS exposure and MV generation, further promoting the procoagulant activity of RBCs [19]. Importantly, association of prothrombotic complications with procoagulant activity of RBCs has been established for cancer patients [18], suggesting that AgNP may present additional thrombotic risk on the cancer patients.

Here, we investigated whether AgNP could affect the procoagulant activity of RBCs, with respect to PS exposure, ROS generation and intracellular calcium increase. The relevance of AgNP-induced procoagulant activity of RBCs to in vivo thrombosis was examined using a rat venous thrombosis model. In an effort to address the thrombotic risk of AgNP in the population susceptible to thrombosis, we examined on the procoagulant effects of AgNP on freshly isolated RBCs from leukemia patients and the synergy with a chemotherapeutic drug (paclitaxel) with thrombotic effects of its own was assessed.

## Results

### Characterization of AgNP and its cellular uptake by RBCs

**Fig. 1:**
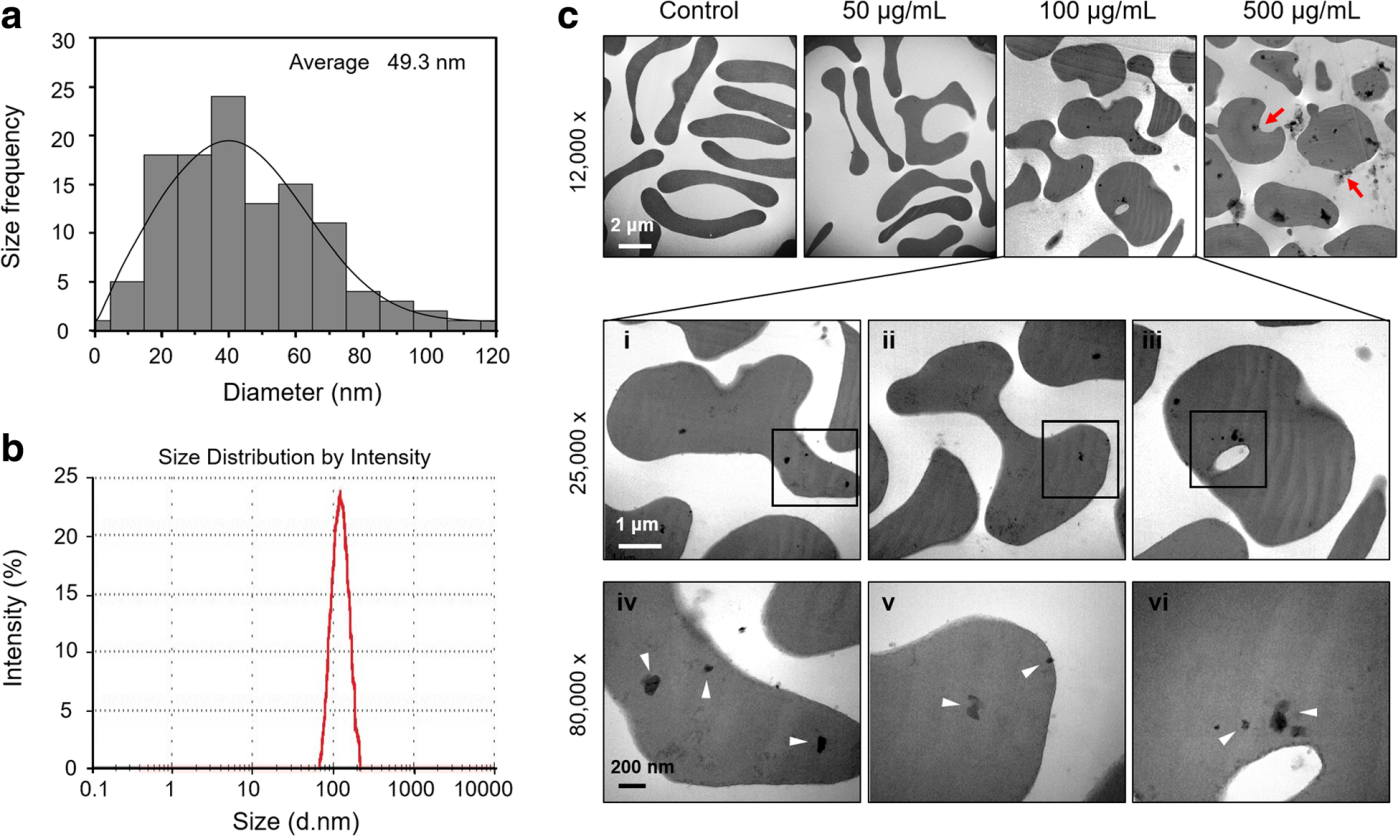
Characterization of AgNP and its cellular uptake by RBCs. **a** The size distribution histogram generated using SEM images showed AgNP of size between 0 and 100 nm with an average of 49.3 nm. Values are calculated from randomly measuring 118 AgNP taken by SEM. **b** Particle size distribution of AgNP in saline suspension used for intravenous injection showed particles of diameter ranging from 32.7 nm to 255 nm, with a peak of distribution at 125.4 nm. The results indicated the presence of AgNP agglomerates in saline. **c** Cellular uptake of various concentrations of AgNP by RBCs were observed using TEM after 4 h treatment. The red arrows indicated cell disruption and lysis at 500 μg/mL of AgNP. Magnifications were 25,000 (**c**, i- iii) and 80,000 (**c**, iv-vi) for 100 μg/mL AgNP. And the white arrows indicated AgNP uptaken by RBCs (*n* = 4)

The size distribution of silver nanoparticles (AgNP) employed in our study was characterized using scanning electron microscopy (SEM). The majority of AgNP were less than 100 nm in size, and the calculated average particle diameter was approximately 49.3 nm (Fig. 1a). The peak size by intensity frequency of AgNP was 125.4 nm (Fig. 1b). And the zeta potential of AgNP was − 32.07 mV at pH 7.4. Ionic silver was separated and quantified using inductively coupled plasma mass spectrometry (ICP-MS) after ultracentrifuge (13,000 rpm for 10 min) and dialysis (24 h), and negligible (< 0.01%) ionic silver was detected. Furthermore, such content of ionic silver was examined with no ability of PS exposure after 4 or 24 h treatment (Additional file 1: Figure S1). The physiochemical properties of AgNP as well as AgMP were summarized in Additional file 2: Table S1. To examine whether AgNP may cross cell membranes of RBCs, we determined the cellular uptake of AgNP by RBCs using transmission electron microscopy (TEM). TEM images showed the cellular uptake of AgNP by RBCs accompanied by morphological alterations, which was in a concentration dependent fashion. Compared to control, AgNP-treated cells showed clear structural alteration at 100 μg/mL and at higher concentration of 500 μg/mL severely damaged cell membranes were observed (Fig. 1c), indicating a potential toxic effect of AgNP on RBCs.

### A pilot study for the following study in vitro

**Fig. 2:**
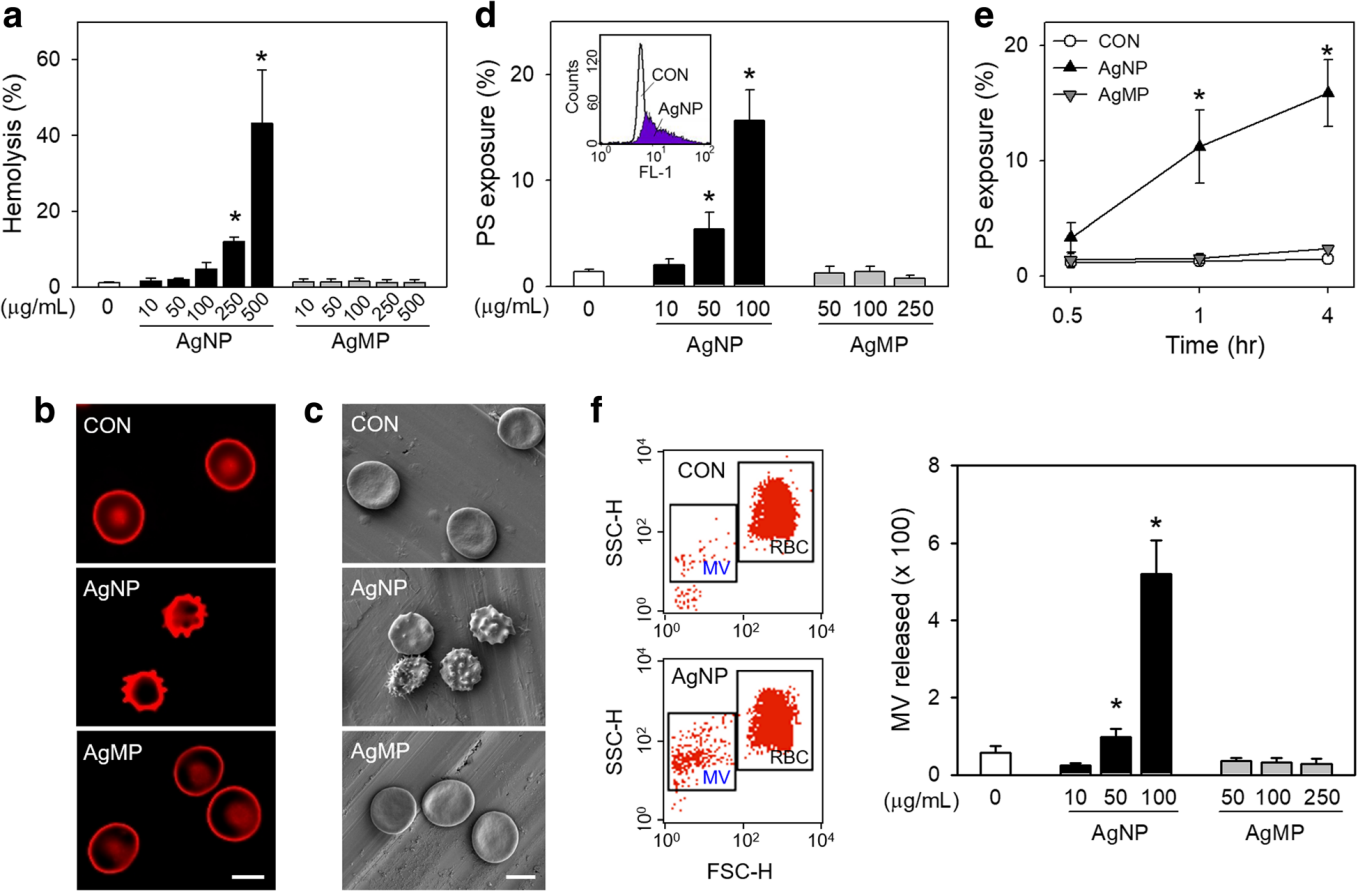
Comparison of effects of AgNP and AgMP on human RBCs. After RBCs were treated with distilled water (DW; control), various concentrations of AgNP and AgMP for 4 h, (**a**) hemolysis was evaluated at 540 nm (*n* = 4). **b, c** RBCs were treated with 100 μg/mL of AgNP and AgMP for 4 h, and examined using confocal microscope (*n* = 4) and scanning electron microscope (*n* = 4), respectively. **d** Concentration- (*n* = 8) and (**e**) time- dependent increase of PS exposure (*n* = 4) as well as (**f**) MV generation by AgNP and AgMP were all measured using flow cytometry (n = 8). Values are mean ± S.E. of 4-8 independent experiments, * represents significant differences from control group (*p* < 0.05). Scale bars (**b**, **c**): 5 μm

A preliminary experiment to find the concentrations with no hemolysis was done prior to the main study. Hemolysis was detected spectrophotometrically at various concentrations of AgNP (Fig. 2a). AgNP induced approximately 11.9 and 43.1% hemolysis at the high concentrations (for 250 and 500 μg/mL, respectively), which was in line with the TEM images (Fig. 1b), indicating that AgNP can induce hemolysis at high concentrations of ≥ 250 μg/mL. As opposed to AgNP, silver microparticles (AgMP) did not induce hemolysis even at high concentrations up to 500 μg/mL, reflecting that size did matter for hemolytic effects of Ag particles.

### Phosphatidylserine (PS) exposure of RBCs and microvesicles (MV) generation at non-hemolytic concentrations

While lower concentrations of AgNP (≤ 100 μg/mL) did not induce hemolysis, TEM observation indicated the cellular uptake of AgNP, suggesting that non-hemolytic effects might be induced in RBCs by AgNP at low doses. Firstly, we investigated the procoagulant activity of RBCs, which is critical to the development of venous thrombosis. Confocal microscopy and SEM observation revealed that the exposure to AgNP induced the occurrence of spiny RBCs, namely echinocytes at 100 μg/mL (4 h) while AgMP was without effects (Fig. 2b, c). A lower effective level could be observed after a longer exposure time compared to the effective level of 50 μg/mL for 4 h incubation with AgNP. As shown in Additional file 3: Figure S2, at 24 h, the effective level of AgNP was 1 μg/mL with around 30% PS exposure, which was 100 times less than that observed at 4 h. It is well known that loss of phospholipid asymmetry is attributable to morphological change in RBCs. During this process, PS can be externalized to outer membrane and membrane surface is lost as MV is generated, which are closely related to procoagulant activity of RBCs [20]. Indeed, in flow cytometry analysis, PS exposure increased in concentration- and time- dependent manners after the exposure to AgNP (Fig. 2d, e). MV generation was also observed upon the exposure to AgNP (Fig. 2f). In contrast to AgNP, even high concentrations of AgMP did not induce any effects on RBCs including hemolysis, shape change, PS exposure or MV generation (Fig. 2).

### Effects of AgNP on oxidative stress and intracellular calcium in human RBCs

**Fig. 3:**
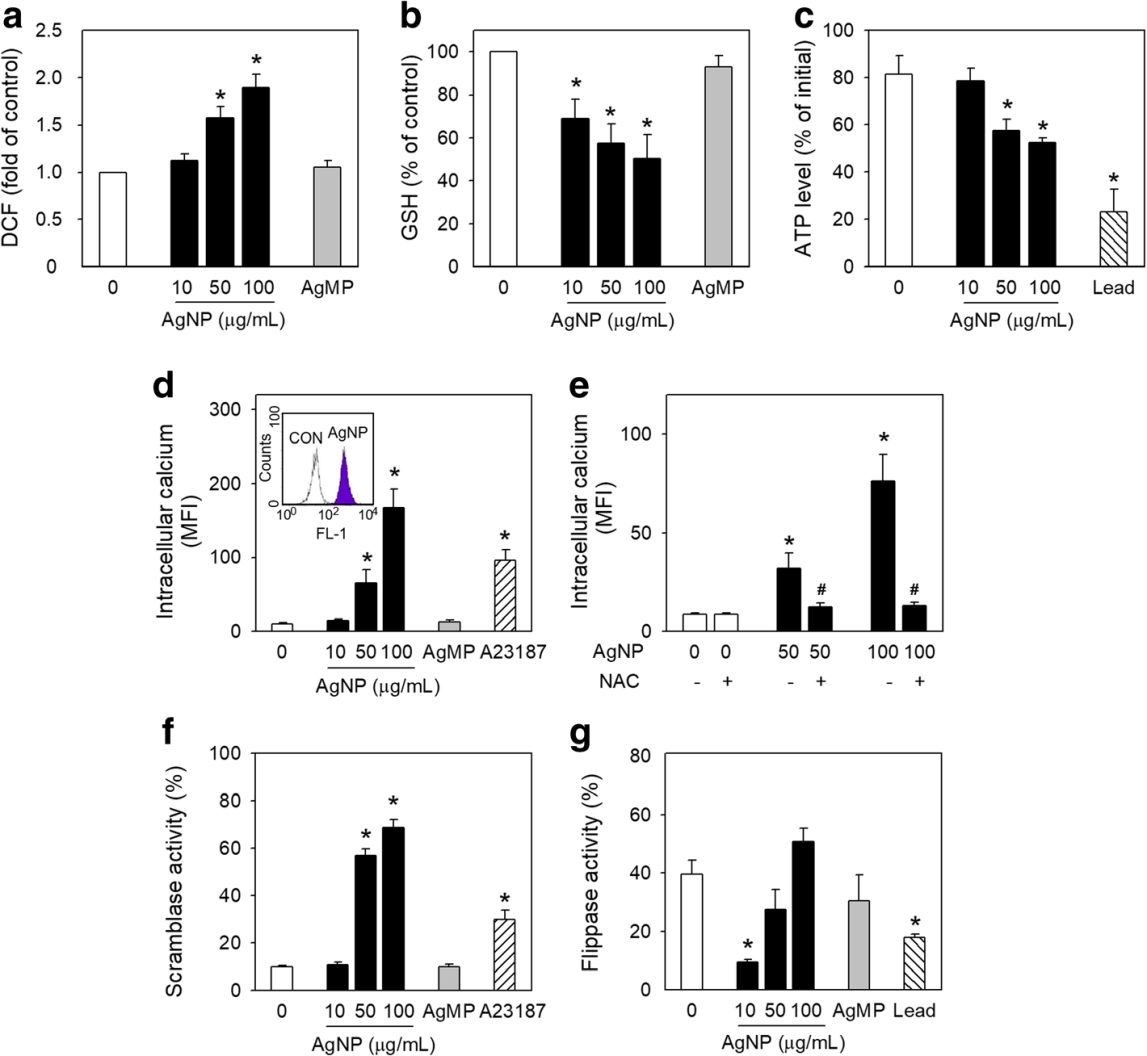
Effects of AgNP on oxidative stress and intracellular calcium in human RBCs. After RBCs were treated with distilled water (DW; control), various concentrations of AgNP and AgMP for 4 h, (**a**) ROS generation (*n* = 6) and (**b**) intracellular GSH level (*n* = 4) (**c**) ATP level (*n* = 4) were detected after RBCs were treated with AgNP and AgMP for 4 h. **d** Intracellular calcium level (*n* = 6) increased after 4 h exposure to various concentrations of AgNP and AgMP and (**e**) inhibition was employed by 5 min pretreatment with 100 μM N-acetylcysteine (NAC) before 4 h exposure to AgNP (*n* = 5). **f** Scramblase activity and (**g**) flippase activity were determined by measuring C_6_-NBD PC and C_6_-NBD PS translocation, respectively (*n* = 4). Values are mean ± S.E. of 4-6 independent experiments, * represents significant differences from control group (*p* < 0.05), # represents significant differences from AgNP-treated group (*p* < 0.05)

Oxidative stress can induce PS exposure and MV generation in RBCs, which can promote the procoagulant activity of RBCs [18]. AgNP-induced ROS generation in RBCs, as examined by DCF-DA-enhanced fluorescence using flow cytometry, increased in a concentration dependent manner, while no effects were observed in AgMP-exposed RBCs (Fig. 3a), which was in a good accordance with PS exposure and MV generation. Consistently with ROS generation, the representative intracellular antioxidant, glutathione (GSH) [21] and ATP were decreased significantly following the exposure to AgNP (Fig. 3b, c).

It is well established that oxidative stress can cause intracellular calcium increase, which is referred as a key stimulus to induce both PS exposure and MV generation [22]. Notably, intracellular calcium level was increased considerably by AgNP (Fig. 3d), which was abolished by the pretreatment with N-acetylcysteine (NAC), a well-known antioxidant (Fig. 3e). Increased intracellular calcium level can activate scramblase, leading to a bidirectional exchange (scrambling) of membrane phospholipids [23] and outward translocation of PS residing on inner membrane leaflet [24]. Indeed, the activity of scramblase was significantly increased as determined by C_6_-NBD-PC externalization (Fig. 3f). Flippase is another aminophospholipid translocase mediating the inward translocation of PS exposed on outer membrane leaflet [20]. The significant decrease in flippase activity at 10 μg/mL indicated an effect of AgNP on the inner ward phospholipid translocation. However, this effect was not shown at higher concentrations of 50 and 100 μg/mL, which appeared to be from the interference of scramblase activity on outer ward phospholipid translocation. Compared with AgNP-exposed groups, no significant effects in ROS generation, GSH depletion, ATP depletion, intracellular calcium level and scramblase activity were observed in AgMP-exposed groups (Fig. 3).

**Fig. 4:**
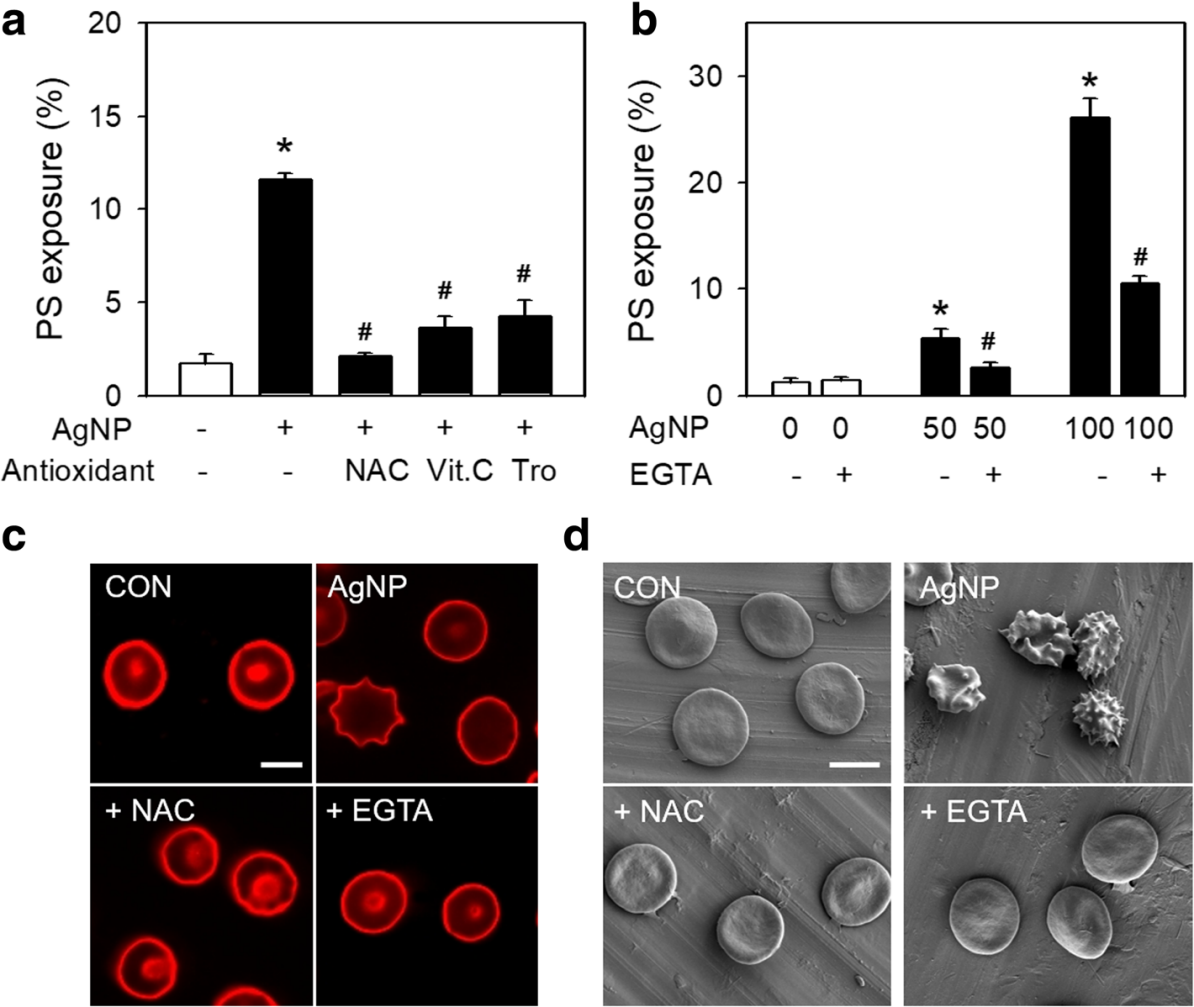
The role of ROS and intracellular calcium on PS exposure and shape change by AgNP. **a** Before treatment with 100 μg/mL AgNP for 4 h, RBCs were preincubated with various antioxidants including 100 μM NAC for 5 min, 0.5 mM Vitamin C (Vit.C) or 1 mM Trolox (Tro) for 15 min, and then PS exposure were determined using flow cytometry (*n* = 4). **b** RBCs were preincubated with 3 mM EGTA for 30 min before treatment with 0, 50 and 100 μg/mL of AgNP for 4 h, and then PS exposure were determined using flow cytometry (*n* = 4). **c**, **d** Before treatment with 100 μg/mL of AgNP for 4 h, RBCs were preincubated with 100 μM NAC for 5 min or 3 mM EGTA for 30 min. Shape change was observed using confocal microscope and scanning electron microscope, respectively (*n* = 4). Values are mean ± S.E. of 4 independent experiments, * represents significant differences from control group (*p* < 0.05), # represents significant differences from AgNP-treated group (*p* < 0.05). Scale bars : 5 μm

To confirm the role of oxidative stress and intracellular calcium increase in AgNP-induced PS exposure, the effects of antioxidants and a calcium chelator, EGTA, were examined. Pretreatment of antioxidants such as NAC, vitamin C and trolox, and EGTA significantly attenuated PS exposure induced by AgNP (Fig. 4a, b), which could be further confirmed by confocal microscopy and SEM (Fig. 4c, d), supporting that oxidative stress and intracellular calcium increase may be key mechanisms underlying AgNP-induced PS exposure.

### Prothrombotic effects of AgNP on human RBCs

**Fig. 5:**
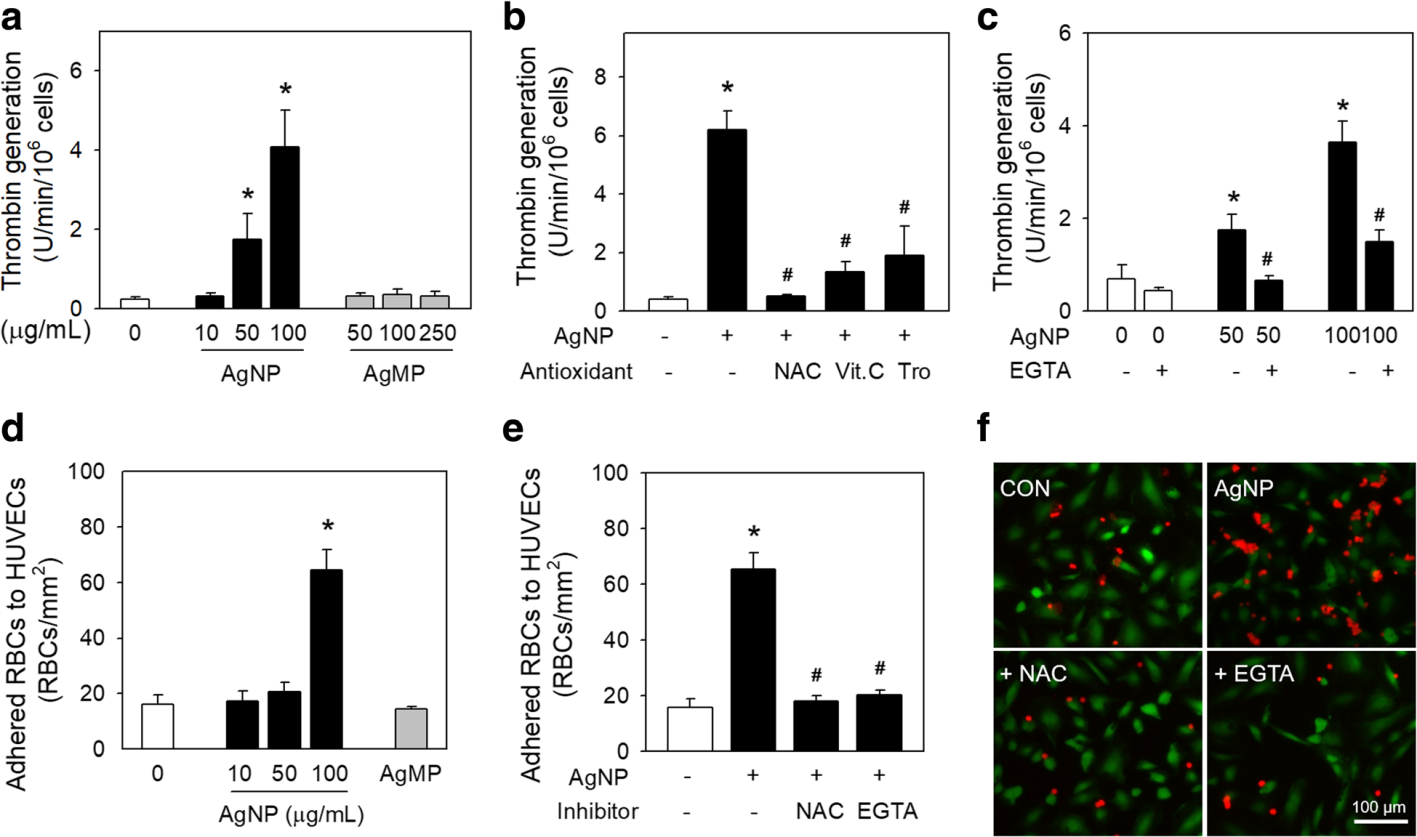
Effects of AgNP on thrombin generation and adhesion of human RBCs to human umbilical vein endothelial cells (HUVECs). Thrombin generation was determined by a prothrombinase assay after RBCs were treated (**a**) with distilled water (DW; control), various concentrations of AgNP and AgMP for 4 h (*n* = 8), (**b**) with 100 μg/mL of AgNP along with preincubation with various antioxidants (*n* = 4) and (**c**) with 0, 50 and 100 μg/mL of AgNP along with preincubation with EGTA (*n* = 4). **d** AgNP- or AgMP-treated RBCs were incubated with HUVECs for 1 h, and then RBCs adhered to HUVECs were counted by microscopic observation (*n* = 4). **e** RBCs were pretreated with NAC or EGTA before AgNP treatment, and RBCs were further incubated with HUVECs for 1 h. And then RBCs adhered to HUVECs were counted by microscopic observation (*n* = 4) or (**f**) examined using fluorescence microscope as described in methods. Red: glycophorin-A-PE for RBC staining; Green: calcein green for HUVECs staining. Values are mean ± S.E. of 4–8 independent experiments. *represents significant differences from control group (*p* < 0.05), # represents significant differences from AgNP-treated group (*p* < 0.05). Scale bars: 100 μm

Prothrombotic effects of AgNP on RBCs were evaluated using examining thrombin generation and RBCs adhesion to human umbilical vein endothelial cells (HUVECs). Procoagulant activity of RBCs results in accelerated thrombin generation, a key step for blood coagulation cascade, and the adhesion of RBCs to vascular wall, which could ultimately promote thrombosis [18]. To investigate whether AgNP-induced PS exposure could promote blood coagulation indeed, thrombin generation by AgNP-exposed RBCs was evaluated. A concentration-dependent increase in thrombin generation was induced by AgNP-exposed RBCs (Fig. 5a), which was significantly inhibited by the pretreatment with antioxidants (Fig. 5b) and EGTA (Fig. 5c). Additionally, adhesion of RBCs to HUVECs was also enhanced by the exposure to AgNP (Fig. 5d). Increased adhesion of RBCs was completely abolished by NAC and EGTA pretreatment (Fig. 5e), which was further confirmed in fluorescence microcopy (Fig. 5f), supporting that exposure of RBCs to AgNP could accelerate coagulation and increase adherence of RBCs to blood vessel, which may ultimately promote thrombosis. These events were all attenuated by antioxidants and a calcium chelator, suggesting the association with oxidative stress and intracellular calcium increase as similar with PS exposure and MV generation. Compared with AgNP-exposed groups, no significant effects on thrombin generation or adherence of RBCs to HUVECs were observed in AgMP-exposed groups (Fig. 5a, d).

### In vivo assessment of AgNP-induced prothrombotic effects

**Fig. 6:**
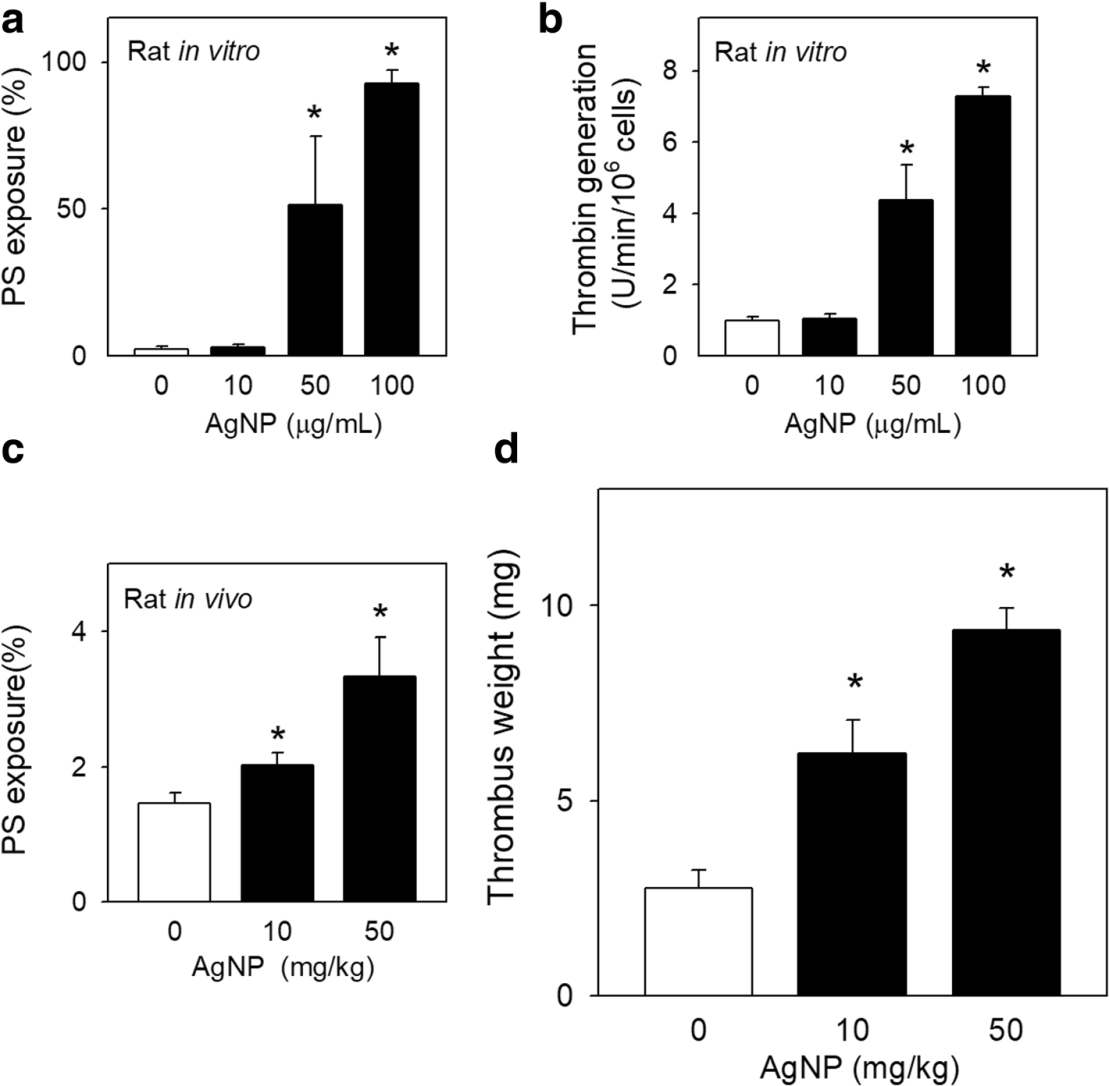
Effects of AgNP exposure on rat venous thrombosis model in vivo. The freshly isolated RBCs from rats were treated with various concentrations of AgNP for 4 h. **a** PS exposure and (**b**) thrombin generation (*n* = 4) were evaluated as described in methods. **c** Blood was collected to determine rat ex vivo PS exposure on RBCs 2 h after intravenous injection of saline (control, *n* = 6) or AgNP (10 mg/kg, *n* = 7 or 50 mg/kg, n = 5). **d** 2 h after intravenous injection of saline (control, n = 6) or AgNP (10 or 50 mg/kg, n = 8 for each dose), thrombus formation was induced by the infusion of thromboplastin in a rat venous thrombosis model. Values are mean ± S.E. of 4–8 independent experiments, * represents significant differences from control group (*p* < 0.05)

Effect of AgNP exposure on thrombosis was further evaluated in rat venous thrombosis model *in vivo*. Firstly, it was confirmed that AgNP exposure led to increased PS exposure (Fig. 6a) and thrombin generation (Fig. 6b) in freshly isolated rat RBCs in a similar fashion observed in human RBCs (Figs. 2d, 5a). Next, to evaluate the prothrombotic effects of AgNP *in vivo*, PS exposure of RBCs and thrombus formation were assessed after intravascular injection of 0, 10 or 50 mg/kg of AgNP. After the administration with AgNP, blood was collected from rats and PS exposure of isolated RBCs was determined *ex vivo* (Fig. 6c). Exposure to AgNP facilitated PS exposure of isolated RBCs in a concentration-dependent manner, which corresponded well with increased thrombus weight (Fig. 6d), reflecting that AgNP induced the procoagulant activity of RBCs and eventually led to increased venous thrombosis.

### Effects of AgNP on procoagulant activity of RBCs isolated from cancer patients and the synergy with chemotherapeutic agents

**Fig. 7:**
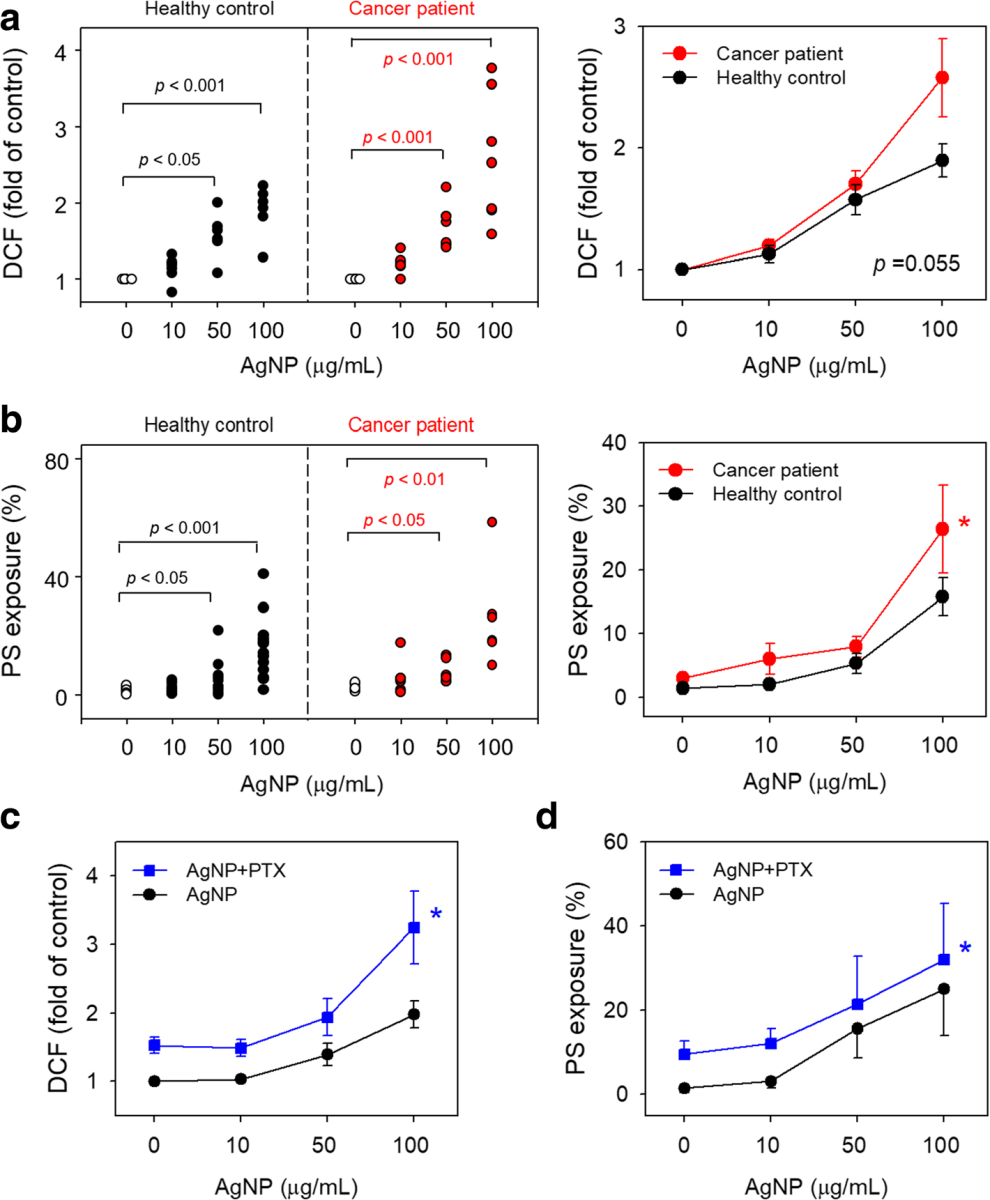
Effects of AgNP on RBCs isolated from cancer patients and its synergy effects with anti-cancer drug. Isolated RBCs from healthy control (black circle, *n* = 8) or from cancer patients (red circle, n = 6) were treated with distilled water (DW; control), various concentrations of AgNP for 4 h, and then (**a**) ROS generation and (**b**) PS exposure were examined using flow cytometry. (**a, b,** left) Dot plots showed individual assessment and (**a, b,** right) line graphs showed overall comparative evaluation between healthy controls (black line) and cancer patients (red line). AgNP alone group (black circle) and paclitaxel co-treated group (blue square) were compared by measurement of (**c**) ROS generation (*n* = 4) and (**d**) PS exposure (*n* = 4). Paclitaxel (25 μM, 15 min) was added after AgNP treatment for 4 h. Values are mean ± S.E. of 4–8 independent experiments, * represents significant differences from AgNP alone group (*p* < 0.05)

AgNP have been applied to the management of cancer for therapeutic inventions and diagnosis, but cancer itself, is a well-known risk factor of thrombosis [14, 15, 25, 26]. RBCs isolated from the patients with leukemia were examined for AgNP-induced procoagulant activity. As a result, the exposure to AgNP induced ROS generation and PS exposure in the RBCs isolated from cancer patients in a concentration dependent manner, which was higher in the degree and lower in the effective concentrations when compared with RBCs from healthy controls (Fig. 7a, b).

Chemotherapeutic drugs to treat cancers also have procoagulant effects [27, 28] and have been previously reported to contribute to increased thrombosis in cancer patients [29]. We investigated whether AgNP may have synergy effects on the procoagulant effects of paclitaxel (PTX), a well-known chemotherapeutic drug which can induce procoagulant activity of RBCs [27]. As shown in Figure 7c and d, the combined exposure to PTX and AgNP significantly increased ROS generation and PS exposure in RBCs compared with those treated with AgNP alone or PTX alone.

## Discussion

**Fig. 8:**
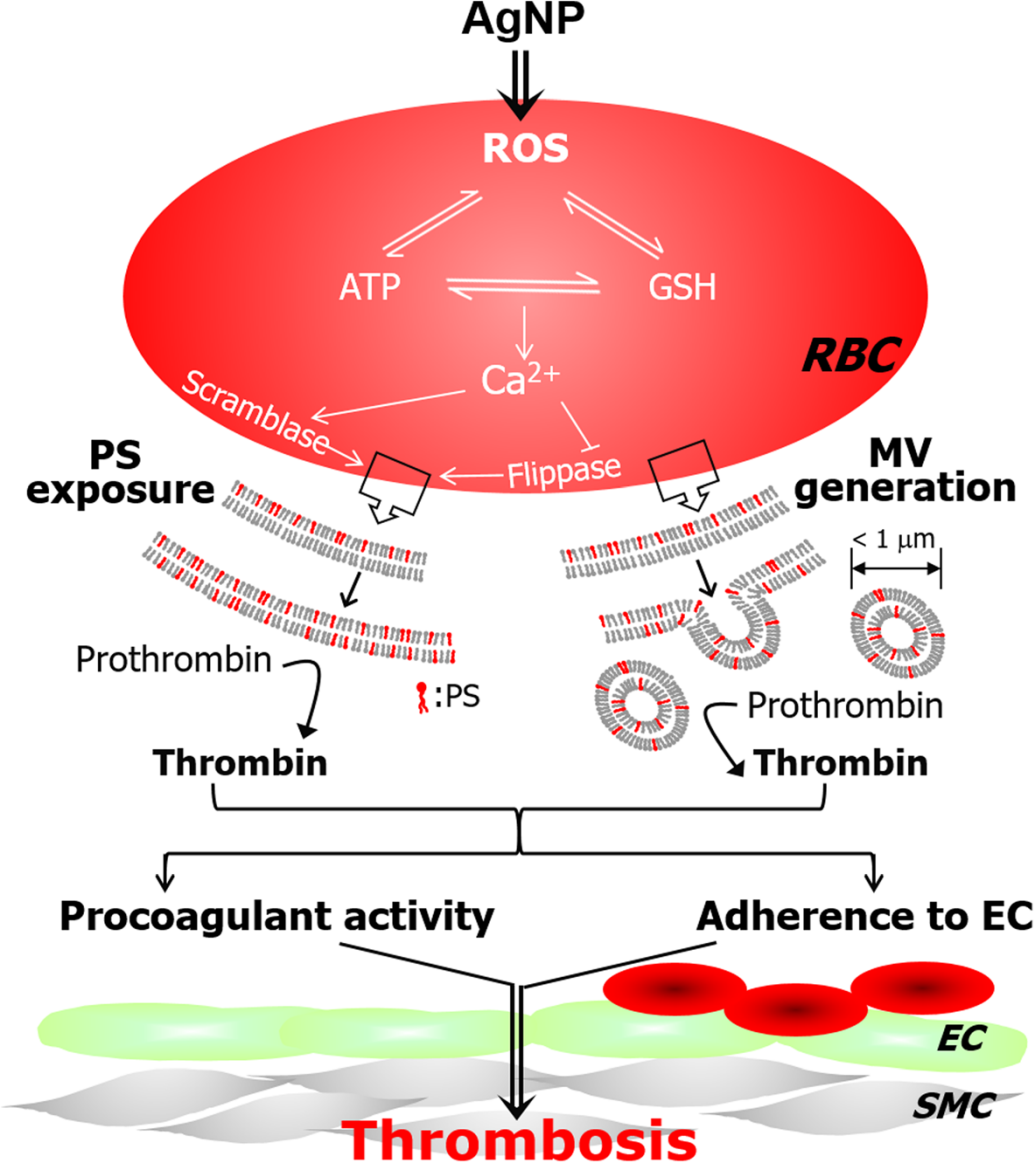
| Suggested mechanism for the procoagulant activity of red blood cells by AgNP

Here, we demonstrated that silver nanoparticles (AgNP) (of sizes less than 100 nm), but not silver microparticles (AgMP), can promote phosphatidylserine (PS) exposure and microvesicles (MV) generation in freshly isolated human red blood cells (RBCs), mainly through reactive oxygen species (ROS) generation and intracellular calcium increase. AgNP-induced procoagulant activity of RBCs was observed at non-hemolytic concentrations and more importantly, confirmed in rat venous thrombosis model *in vivo* where the exposure to AgNP led to increased PS exposure and promoted thrombosis supporting the relevance of our findings to real *in vivo* states (Fig 8). Of note, RBCs of cancer patients were more sensitive to the procoagulant effects of AgNP and AgNP manifested the synergy with a chemotherapeutic agent, paclitaxel in promoting procoagulant activity of RBCs, suggesting that a caution shall be taken for the application of AgNP to the management of cancer patients.

Toxicity of AgNP has been investigated in many studies, including cytotoxicity of AgNP in various cells *in vitro* [4, 6, 30], and assessment of bio-distribution, elimination, and accumulation of AgNP in organs and potential organ toxicity associated with AgNP exposure [7, 31]. Considering the direct contact of blood cells with AgNP entering the body during circulation, potential cardiovascular toxicity of AgNP shall not be ruled out. However, few studies have addressed the mechanism underlying AgNP-induced toxicity on RBCs in relation to pathophysiological effects. Previous studies have focused on the direct toxicity of AgNP on the structural integrity of RBCs or early events such as hemolysis, oxidative stress and membrane damage [11, 32, 33] without extending them into the mechanisms leading to diseases. Here, we demonstrated that AgNP may promote thrombosis through procoagulant activity of RBCs providing an important piece of clue for the potential cardiovascular toxicity of AgNP.

In RBCs, toxicological effects of AgNP have been investigated regarding the hemolysis employing extremely high concentration ranges (up to 500 μg/mL) of AgNP [11, 32]. We could also observe a significant hemolysis at high concentrations of AgNP (from 250 μg/mL to 500 μg/mL), which matched well with previous studies of hemolysis. Actually, this range is too high and raises questions on its clinical relevance. Of note, we observed that AgNP can induce PS exposure and procoagulant activity at non-hemolytic concentrations as low as 50 μg/mL after 4 h exposure, demonstrating that AgNP can cause adverse effects on cardiovascular system at non-hemolytic ranges. This concentration range of AgNP is comparable with those (25 ~ 400 μg/mL) used in the previous literatures on AgNP toxicity against human peripheral blood cells or lung cells [6, 7]. Furthermore, we also observed that a prolonged exposure (24 hr) could significantly lower the effective level to 1 μg/mL as shown in Additional file 3: Figure S2, indicating a more potent toxicity could be expected after a longer exposure. Currently, reports on the toxicity of AgNP in vivo are often controversial and inconsistent. In an attempt to demonstrate AgNP toxicity in vivo*,* diverse exposure routes, such as oral exposure, inhalation, intraperitoneal and intravenous, in the settings of single or multiple exposure and with various AgNP differed in size, surface charge and surface-coating substances have been explored in many experimental animal species. While oral administration of AgNP unanimously showed no obvious toxicity in acute or subacute studies [34–36], intravenous (i.v.) exposure to AgNPs was not without toxicity. Previous studies observed spleen and liver toxicity of AgNP in mice after 10 mg/kg i.v. administration, and liver and hematological toxicity in rats after i.v. injection of 4~40 mg/kg AgNP [37, 38]. Consistently, in our study, AgNP induced thrombosis at ≥ 10 mg/kg by promoting procoagulant activation of RBCs. After i.v. injection of 10 mg/kg AgNP in rats, peak concentration of 5.2 μg/mL was observed at 8 h, which was basically maintained at around 5 μg/mL up to 96 h [39] of which range was similar to our studies in vitro and in vivo.

It has been still controversial that whether the toxic effect is particle-specific or mediated by silver ions dissolved from AgNP. A previous literature suggested released silver ions were the primary cause of AgNP toxicity on *Sphaerium corneum* [40], and the effect of silver ion on RBCs was reported by Sopjani et al. [41]. However, Recordati et al. showed that silver ion and AgNP exhibit distinct patterns of tissue distribution and toxicity, and toxicity of AgNP was attributable to the nanoparticle form [37]. Consistently, we could observe negligible release of silver ion from AgNP and more importantly, found that silver ion did not induce PS exposure shown in Additional file 1: Fig. S1, although the dissolution may differ depending on physiochemical characteristics (e.g. size, shape, surface charge, pH etc.) [42].

ROS and oxidative stress are involved in regulating normal physiology as well as pathophysiological processes. Excessive production of ROS has been well established to orchestrate a series of pathological events such as genotoxicity, inflammation, fibrosis, and carcinogenesis [43, 44]. ROS generation induced by AgNP has been reported to provoke cytotoxicity and genotoxicity in leukemia cells and lung cells [6, 7], indicating that ROS generation may explain the toxicity of AgNP. Indeed, ROS generation played a central role in the procoagulant and prothrombotic effects of AgNP on RBCs. Interestingly, other nanomaterials such as carbon, titanium dioxide (TiO_2_) and manganese oxide (MnO_2_) have been shown to induce oxidative stress triggering signaling cascades that eventually result in increased expression of proinflammatory, fibrotic cytokines and cell damage [21, 43, 45]. In this context, in addition to AgNP, potential effects of other nanomaterials on procoagulant activity of RBCs warrant further attention as well [46].

Recently, nanotechnology has been incorporated into various fields of medical practice. Nanomedicine has emerged as the mainstream in cancer management. Notably, cancer patients are at a hypercoagulable state as for such reasons as advanced stage of diseases, intravascular metastasis, and increased tumor volume [14, 25, 29]. Clinically detectable thrombotic events were found in 15~20% of all cancer patients [14, 15, 25]. Cancer, itself, is a major thrombotic risk factor increasing thrombosis by 4.1 fold and moreover, chemotherapeutic agents heighten it even further by 6.5 fold [15, 26], reflecting the risk of AgNP-use in cancer management. Of critical, exposure to AgNP in combination of chemotherapeutic agent may aggravate thrombotic risks when combined with the hypercoagulable state of cancer patients, which needs further studies in the future.

## Conclusion

Our study demonstrated that AgNP can promote prothrombotic risks by increasing procoagulant activity of RBCs both *in vitro* and *in vivo*. The mechanism underlying the effect appears to involve oxidative stress and reactive oxygen species generation. Of note, procoagulant effects of AgNP were enhanced in RBCs of cancer patients and a significant synergy was observed with a chemotherapeutic agent, paclitaxel in promoting procoagulant activity of RBCs, suggesting that caution should be taken for its use in cancer patients.

## Methods

### Materials

AgNP (silver nanoparticles, < 100 nm particle size, contains 0.2% PVP as dispersant, 99.5% trace metals basis.), AgMP (silver microparticles, 5-8 micro, ≥ 99.9% metals basis), mercury orange, trolox, L-ascorbic acid, glutaldehyde solution and osmium tetroxide, the ATP bioluminescent assay kit, and purified human thrombin were purchased from Sigma Chemical Co. (St. Louis, MO). Phycoerythrin-labeled monoclonal mouse anti-human CD235a and fluorescein-isothiocyanate (FITC)-labeled annexin V (annexin V-FITC) were from BD Pharmingen (San Diego, CA). Fluo-4 acetoxymethyl ester (fluo-4 AM) and the chloromethyl derivative of 2′, 7′-dichlorodihydrofluorescein diacetate (CM-H_2_DCF-DA) were obtained from Life Technologies.1-Palmitoyl-2-[6-[(7-nitro-2-1,3-benzoxadiazol-4-yl)amino]hexanoyl]-snglycero-3-phospho-L-serine (C_6_-NBD-PS) and 1-oleoyl-2-[6-[(7-nitro-2-1,3-benzoxadiazol-4-yl)amino]hexanoyl]-sn-glycero-3-phosphocholine (C_6_-NBD-PC) were from Avanti Polar Lipids (Alabaster, AL). Purified human prothrombin, factor Xa and factor Va were from Hematologic Technologies, Inc. (Essex Junction, VT), and S2238 was from Chromogenix (Milano, Italy). Human umbilical vein endothelial cells (HUVECs) and the endothelial cell growth media (EGM) kit were purchased from Lonza. Human recombinant tissue factor (Recombiplastin) was obtained from Instrumentation Laboratory (Lexington, MA) and thromboplastin (Simplastin Excel) was from Biomerieux (Durham, NC).

### Assessment of characterization of AgNP

Both AgNP and AgMP were purchased from Sigma Aldrich. Preparation of AgNP suspension was carried out according to the methods previously described [12]. AgNP or AgMP powder were dispersed in distilled water as 100 X stock solution (1–50 mg/mL) and sonicated with a probe type sonicator with a maximum output power, 200 Watts (Branson Sonifier, Danbury, CT) for 2 min to prevent particles self-assembly (agglomeration). In addition, prior to every experiment, AgNP suspensions were vigorously vortexed for 30 sec. AgNP we used in the present study were with 0.2% PVP as dispersant (provided by Sigma Aldrich), while no PVP was in AgMP (provided by Sigma Aldrich). Through mathematical calculation, there might be 0.2 μg/mL PVP in 10 μg/mL AgNP-treated group, while 2 μg/mL PVP in 100 μg/mL AgNP-treated group. In order to estimate the effect of PVP in AgNP on PS exposure, we determined the effect of 2 μg/mL PVP alone on PS exposure of RBCs. Results are as follows: Control (1.52 ± 0.90% PS exposure); 2 μg/mL PVP (1.38 ± 1.46% PS exposure). Since PVP had minimal effects, DW (distilled water) was used as a vehicle control.

For the characterization of AgNP, AgNP suspension was dried and was observed with scanning electron microscope (SEM) (ZEISS, MERLIN Compact) to examine the size frequency. A detailed statistical analysis of AgNP was performed by randomly measuring 118 nanoparticles and the procedure was operated by manually outlining the particles from several images taken by SEM. Nanoparticles were used at a concentration of 100 μg/mL in the following determination. The hydrodynamic diameter and the zeta potential of the nanoparticles were measured by dynamic light scattering (DLS-7000) and electrophoretic light scattering (ELS Z-100), respectively. The polydispersity index (PDI) was below 0.3 in all cases. For determination of dissolved silver, ultracentrifuge (13,000 rpm for 10 min) and dialysis (24 hr) were prior to the quantification of Ag. Dialysis experiments were carried out in dialysis bags filled with 5 mL silver nanoparticle dispersion (100 μg/mL) and immersed in 495 mL water. The concentration of silver was < 0.1 μg/L determined by inductively coupled plasma mass spectrometry (ICP-MS) with the limit of detection of 0.1 μg/L. The maximum possible percentage of ionic Ag in silver nanoparticle dispersion was 0.01%.

### Preparation of RBCs

With the approval from the Ethics Committee of Health Service Center at Seoul National University, human blood was obtained from healthy male donors (20-30 years old) using a vacutainer with acid citrate dextrose (ACD) and a 21 gauge needle (Becton Dickinson, U.S.A.) on the day of each experiments. Platelet rich plasma and buffy coat were removed by aspiration after centrifugation at 200 g for 15 min. Packed RBCs were washed 2 times with phosphate buffered saline (PBS: 1.06 mM KH_2_PO_4_, 154 mM NaCl and 2.96 mM Na_2_HPO_4_ at pH 7.4) and once more with Ringer's solution (125 mM NaCl, 5 mM KCl, 1 mM MgSO_4_, 32 mM HEPES, 5 mM glucose, pH 7.4). Washed RBCs were resuspended in TBS buffer to a cell concentration of 5 x 10^7^ cells/mL, and the final CaCl_2_ concentration was adjusted to 1 mM prior to use.

### Observation of cellular uptake of AgNP by RBCs

For detection of cellular uptake of AgNP by RBCs, isolated RBCs were incubated with AgNP for 4 h, and fixed with 2% glutaraldehyde solution in the refrigerator overnight. On the second day, after washing three times with 0.05 M sodium cacodylate buffer every 10 min, cells were post-fixated with 2% osmium tetroxide for 2 h. Before en-bloc staining with 0.5% uranyl acetate for 30 minutes, briefly washing with DW were done twice, and serially dehydrated once with 30, 50, 70, 80, 90% and three time with 100% ethanol. Next, propylene oxide was used for transition every 10 min for 2 times, and infiltration were done once with propylene oxide and spurr’s resin (1:1) for 2 h, then with only spurr’s resin in the desiccator overnight. On the third day, infiltration was completed with newly spurr’s resin for 2 h in the desiccator, then samples were kept in the 70°C oven overnight for polymerization. Finally, samples were observed under TEM (JEOL, JEM 1010).

### Flow cytometric analysis of phosphatidylserine (PS) exposure and microvesicles (MV) generation

Annexin V-FITC was used as a marker for PS detection, whereas anti-glycophorin A-PE was used as an identifier of RBCs. Negative controls for annexin V binding were stained with annexin V-FITC in the presence of 2.5 mM EDTA instead of 2.5 mM CaCl_2_. Samples were analyzed on the flow cytometer FACS Calibur (Becton Dickinson, U.S.A.) equipped with an argon-ion laser emitting at 488 nm. The light scatter and fluorescence channels were set on a log scale. Data from 5,000 events were collected and analyzed using Cell Quest Pro software. MV were identified on the basis of forward scatter characteristics after calibration by 1 μM standard beads. The extent of hemolysis was determined spectrophotometrically at 540 nm.

### Microscopic observation using confocal and scanning electron microscopy (SEM)

For confocal microscopy, 500 μL RBCs suspension was added and attached for 1 h to a four-chambered coverslip (Lab-Tek; Nalge Nunc Inc., Naperville, IL). After washing the coverslip once with Ringer’s solution containing 2% BSA, RBCs were then incubated with a vehicle control (DW in Ringer’s solution), AgNP or AgMP (dissolved in Ringer’s solution). After incubation, the coverslips were washed once again with Ringer’s solution and then, RBCs were stained with Ringer’s solution containing anti–glycophorin-A–PE for 30 min. Ultimately, samples were washed once and resuspended to be observed using confocal microscopy equipped with an argon laser (Leica, Wetzlar, Germany). Excitation and emission filters were set at 488 nm and 550–600 nm, respectively. For SEM observation, after fixation with 2% glutaraldehyde solution for 1 h at 4°C, the RBCs were centrifuged and washed three times with PBS, followed by post-fixation with 1% osmium tetroxide for 30 min at room temperature in the hood. After washing with PBS twice, the samples were dehydrated serially with 50, 70, 80, 90, and 100% ethanol. After drying and coating with gold, the images were observed on a SEM.

### Measurement of reactive oxygen species (ROS) and intracellular glutathion (GSH) level

To detect ROS generation, RBCs were preincubated with 5 μM CM-H_2_DCF-DA for 30 min at 37°C. After washing with Ringer’s solution containing 1 mM CaCl_2_ to remove excess CM-H_2_DCF-DA, RBCs were incubated with AgNP or AgMP. The aliquots were diluted and the fluorescence of intracellular DCF was measured using flow cytometry. GSH level in RBCs was measured according to the method described by Jose Enrique O’Connor et al [47]. After incubated with vehicles (DW), AgNP or AgMP, RBCs were treated with 40 μM mercury orange (dissolved in acetone) and then incubated in ice for 5 min. After that, cells were washed and resuspended with cold Ringer's solution, and then diluted with cold Ringer’s solution to be analyzed with an FACS Calibur flow cytometer (BD Biosciences).

### Measurement of intracellular ATP and calcium levels

Samples were washed and resuspended in Ringer’s solution containing 1 mM CaCl_2_. The aliquot was mixed vigorously with TAE buffer (100 mM Tris-acetate, 2 mM EDTA, pH 7.8) containing 10% trichloroacetic acid, and then cooled in ice for 20 min. The sample was centrifuged and the aliquot of resultant supernatant was mixed with cold TAE buffer. The intracellular ATP level was measured by luciferin/luciferase assay in Luminoskan (Labsystems, Franklin, Massachusetts) using an ATP assay kit. We calculated the ATP concentrations based on the ATP standard curve. To detect intracellular calcium increase, RBCs were loaded with 3 μM Fluo-4 AM (Molecular Probes, Eugene, OR, USA) for 1h at 37°C in the dark. After excess Fluo-4 AM was removed, AgNP or AgMP were treated to RBCs. The aliquots were diluted and analyzed in flow cytometer. Data from 5,000 events were collected and analyzed using Cell Quest Pro software (Becton Dickinson).

### Phospholipid translocation measurement

RBCs (5 x 10^7^ cells/mL) were incubated with AgNP or AgMP for 30 min at 37 °C and then loaded with 0.5 μM C_6_-NBD-PC and C_6_-NBD-PS. Aliquots from the cell suspension were removed at the indicated time intervals and placed on ice for 20 min in the presence or absence of 1 % bovine serum albumin (BSA). Pellets obtained after 1 min of centrifugation at 12,000 g were lyzed in 1% Triton X-100, and the fluorescence intensities were measured (ex 485 nm, em 535 nm). The amount of internalized probe was determined by comparing the fluorescence intensity associated with the cells before and after back-extraction.

### Prothrombinase assay

After incubation with AgNP or AgMP for 4 h, samples were incubated with 5 nM factor Xa and 10 nM factor Va in Tyrode buffer (134 mM NaCl, 10 mM HEPES, 5 mM glucose, 2.9 mM KCl, 1 mM MgCl_2_, 12 mM NaHCO_3_, 0.34 mM Na_2_HPO_4_, 0.3% BSA, and 2 mM CaCl_2_ at pH 7.4) for 3 min at 37 °C. Thrombin formation was initiated by addition of 2 μM prothrombin. Exactly 3 min after the addition of prothrombin, an aliquot of the suspension was transferred to a tube containing stop buffer (50 mM Tris-HCl, 120 mM NaCl, and 2 mM EDTA at pH 7.9). Thrombin activity was determined using the chromogenic substrate S2238 (chromogenic substrate for thrombin; Chromogenix, Milano, Italy). We calculated the rate of thrombin formation from the change in absorbance at 405 nm using a calibration curve generated with active-site–titrated thrombin.

### Adherence of RBCs to human umbilical vein endothelial cells (HUVECs)

The HUVECs (6-8 passages) were maintained in the endothelial cell growth media kit at 37°C in a 95% air/5 % CO_2_ incubator. Endothelial cells (1x10^5^ cells) were seeded into 25T flask for 5 days, AgNP or AgMP-treated RBCs were washed once and resuspended in EBM-2 to a cell concentration of 5x10^7^ cells/mL. After HUVEC was washed twice with EBM-2, AgNP or AgMP-exposed RBCs were layered onto confluent HUVEC monolayer and incubated for 60 min at 37°C. After the incubation, the flask was rinsed 3 times with EBM-2 to remove nonadherent RBCs. The number of adherent RBCs was counted on light microscope. The experiments were performed in triplicate and 25 fields were selected randomly to count RBCs number.

### Fluorescence observation of adhered RBCs to HUVECs

Endothelial cells (2 x 10^4^ cells) were seeded into 4-well-chamber for 2 days and stained with calcein green for 20 min. AgNP or AgMP-treated RBCs were washed once and resuspended in EBM-2 to a cell concentration of 5x10^7^ cells/mL. After HUVECs were washed twice with EBM-2, AgNP or AgMP-exposed RBCs were layered onto confluent HUVEC monolayer and incubated for 60 min at 37°C. After the incubation, the chambers were rinsed once with EBM-2 to remove nonadherent RBCs, and glycophorin A-PE were added for staining RBCs. Adhered RBCs to HUVECs were observed using fluorescent microscopy.

### In vitro assessment of PS exposure and procoagulant activity in rat RBCs

Male Sprgue-Dawley rats (300-400g) were anesthetized with urethane (1.25 g/kg, i.p.). Briefly, citrated whole blood (3.8% sodium citrate) was withdrawn from abdominal aorta and RBCs were isolated as human RBCs preparation. Isolated RBCs were further incubated with AgNP for 4 h, then PS exposure and procoagulant activity were determined mentioned above.

### In vivo experiments

Male Sprague-Dawley rats (300-400 g) were anesthetized with urethane (1.25 g/kg, i.p.). 2 h after intravascular injection of AgNP (10 or 50 mg/kg), whole blood (3.8% sodium citrate) was collected from abdominal aorta, and PS exposure of RBCs was determined using flow cytometry. Thrombus formation was induced by stasis combined with hypercoagulability. The abdomen was surgically opened, and the vena cava was exposed after careful dissection. Two loose cotton threads were prepared 16 mm apart around the vena cava. All side branches were ligated tightly with cotton threads. 2 h after the intravenous injection of AgNP (10 or 50 mg/kg) into a left femoral vein, 1000-fold diluted thromboplastin was infused for 1 min to induce thrombus formation. Stasis was initiated by tightening the two threads, first the proximal and the distal thereafter. The abdominal cavity was provisionally closed, and blood stasis was maintained for 15 min. After the abdomen was re-opened, the ligated venous segment was excised and opened longitudinally to remove the thrombus. The isolated thrombus was blotted of excess blood and immediately weighed.

### Experiments using RBCs from cancer patients

With an approval from the Ethics Committee of Health Service Center at Seoul National University Hospital, human blood was obtained from cancer patient male donors with leukemia and healthy control male donors using a vacutainer with acid citrate dextrose and a 21-gauge needle on the day of each experiment. RBCs preparation, measurement of ROS generation and PS exposure were done as described earlier.

### Statistical analysis

The means and standard errors of means were calculated for all treatment groups. The data were subjected to two-way analysis of variance followed by Duncan’s multiple range test or student t test to determine which means were significantly different from the control. In all cases, a *p* value of < .05 was used to determine significant differences.

## Additional files

Additional file 1: **Figure S1.** PS exposure of human red blood cells induced by ionic silver (0.1 μM). (TIF 208 kb)

Additional file 2: **Table S1.** Summary of physicochemical properties of AgNP and AgMP. (TIF 314 kb)

Additional file 3: **Figure S2.** PS exposure of human red blood cells induced by low concentrations of AgNP after a prolonged exposure. (TIF 232 kb)

## References

[1] Wei L (2015). Silver nanoparticles: synthesis, properties, and therapeutic applications. Drug Discov Today.

[2] Ong C (2013). Silver nanoparticles in cancer: therapeutic efficacy and toxicity. Curr Med Chem.

[3] Martínez-Gutierrez F (2012). Antibacterial activity, inflammatory response, coagulation and cytotoxicity effects of silver nanoparticles. Nanomedicine: NBM.

[4] Braydich-Stolle L (2005). In vitro cytotoxicity of nanoparticles in mammalian germline stem cells. Toxicol Sci.

[5] Ivask A (2013). Toxicity mechanisms in Escherichia coli vary for silver nanoparticles and differ from ionic silver. ACS Nano.

[6] AshaRani P (2008). Cytotoxicity and genotoxicity of silver nanoparticles in human cells. ACS Nano.

[7] Ghosh M (2012). In vitro and in vivo genotoxicity of silver nanoparticles. Mutat Res Genet Toxicol Environ Mutagen.

[8] Tang J (2009). Distribution, translocation and accumulation of silver nanoparticles in rats. J Nanosci Nanotechnol.

[9] Nemmar A (2002). Passage of inhaled particles into the blood circulation in humans. Circulation.

[10] Takenaka S (2001). Pulmonary and systemic distribution of inhaled ultrafine silver particles in rats. Environ Health Perspect.

[11] Kim MJ, Shin S (2014). Toxic effects of silver nanoparticles and nanowires on erythrocyte rheology. Food Chem Toxicol.

[12] Jun E-A (2011). Silver nanoparticles enhance thrombus formation through increased platelet aggregation and procoagulant activity. Nanotoxicology.

[13] Greulich C (2011). Cell type-specific responses of peripheral blood mononuclear cells to silver nanoparticles. Acta Biomater.

[14] Lee AY, Levine MN (2003). Venous thromboembolism and cancer: risks and outcomes. Circulation.

[15] Heit JA (2000). Risk factors for deep vein thrombosis and pulmonary embolism: a population-based case-control study. Arch Intern Med.

[16] Lim K-M (2010). Low-level mercury can enhance procoagulant activity of erythrocytes: a new contributing factor for mercury-related thrombotic disease. Environ Health Perspect.

[17] Shin J-H (2007). Lead-induced procoagulant activation of erythrocytes through phosphatidylserine exposure may lead to thrombotic diseases. Chem Res Toxicol.

[18] Kim K (2015). High-dose vitamin C injection to cancer patients may promote thrombosis through procoagulant activation of erythrocytes. Toxicol Sci.

[19] Mohanty JG, Nagababu E, Rifkind JM. Red blood cell oxidative stress impairs oxygen delivery and induces red blood cell aging. Front Physiol. 2014;5. 10.3389/fphys.2014.00084 PMC3937982 24616707

[20] Robert F.A. Zwaal and Alan J. Schroit. Pathophysiologic Implications of Membrane Phospholipid Asymmetry in Blood Cells. Blood. 1997;89:1121-32. 9028933

[21] Xia T (2006). Comparison of the abilities of ambient and manufactured nanoparticles to induce cellular toxicity according to an oxidative stress paradigm. Nano Lett.

[22] DeJong K (2006). High calcium requirement for phosphatidylserine exposure in sickle cell disease. Blood.

[23] Suzuki J (2013). Calcium-dependent phospholipid scramblase activity of TMEM16 protein family members. J Biol Chem.

[24] Weiss E, Rees DC, Gibson JS (2011). Role of calcium in phosphatidylserine externalisation in red blood cells from sickle cell patients. Anemia.

[25] Caine GJ (2002). The hypercoagulable state of malignancy: pathogenesis and current debate. Neoplasia.

[26] Piatek C, O'connell CL, Liebman HA (2012). Treating venous thromboembolism in patients with cancer. Expert Rev Hematol.

[27] Lang PA (2006). Stimulation of erythrocyte phosphatidylserine exposure by paclitaxel. Cell Physiol Biochem.

[28] Briglia M (2015). Edelfosine induced suicidal death of human erythrocytes. Cell Physiol Biochem.

[29] Levine MN (1988). The thrombogenic effect of anticancer drug therapy in women with stage II breast cancer. N Engl J Med.

[30] Xiu Z-m (2012). Negligible particle-specific antibacterial activity of silver nanoparticles. Nano Lett.

[31] van der Zande M (2012). Distribution, elimination, and toxicity of silver nanoparticles and silver ions in rats after 28-day oral exposure. ACS Nano.

[32] Asharani P (2010). Investigations on the structural damage in human erythrocytes exposed to silver, gold, and platinum nanoparticles. Adv Funct Mater.

[33] Chen LQ (2015). Nanotoxicity of silver nanoparticles to red blood cells: size dependent adsorption, uptake, and hemolytic activity. Chem Res Toxicol.

[34] Kim YS (2008). Twenty-eight-day oral toxicity, genotoxicity, and gender-related tissue distribution of silver nanoparticles in Sprague-Dawley rats. Inhal Toxicol.

[35] Hong J-S (2014). Combined repeated-dose toxicity study of silver nanoparticles with the reproduction/developmental toxicity screening test. Nanotoxicology.

[36] Smock KJ (2014). Assessment of orally dosed commercial silver nanoparticles on human ex vivo platelet aggregation. Nanotoxicology.

[37] Recordati C (2015). Tissue distribution and acute toxicity of silver after single intravenous administration in mice: nano-specific and size-dependent effects. Part Fibre Toxicol.

[38] Tiwari DK, Jin T, Behari J (2011). Dose-dependent in-vivo toxicity assessment of silver nanoparticle in Wistar rats. Toxicol Mech Methods.

[39] Park K (2011). Bioavailability and toxicokinetics of citrate-coated silver nanoparticles in rats. Arch Pharm Res.

[40] Völker C (2015). Toxicity of silver nanoparticles and ionic silver: comparison of adverse effects and potential toxicity mechanisms in the freshwater clam Sphaerium corneum. Nanotoxicology.

[41] Sopjani M (2009). Silver ion-induced suicidal erythrocyte death. J Appl Toxicol.

[42] Utembe W (2015). Dissolution and biodurability: important parameters needed for risk assessment of nanomaterials. Part Fibre Toxicol..

[43] Oberdörster G, Oberdörster E, Oberdörster J (2005). Nanotoxicology: an emerging discipline evolving from studies of ultrafine particles. Environ Health Perspect.

[44] Scherz-Shouval R, Elazar Z (2011). Regulation of autophagy by ROS: physiology and pathology. Trends Biochem Sci.

[45] Fan K (2018). In vivo guiding nitrogen-doped carbon nanozyme for tumor catalytic therapy. Nat Commun.

[46] Nel A (2006). Toxic potential of materials at the nanolevel. Science.

[47] O'Connor JE (1988). A flow cytometric assay for intracellular nonprotein thiols using mercury orange. Cytometry A.

