# Supplementary figures and images for: Silver nanoparticles promote procoagulant activity of red blood cells: a potential risk of thrombosis in susceptible population
Source: Part Fibre Toxicol. 2019 Feb 14;16:9. doi: 10.1186/s12989-019-0292-6 (PMC6376700; doi:10.1186/s12989-019-0292-6)

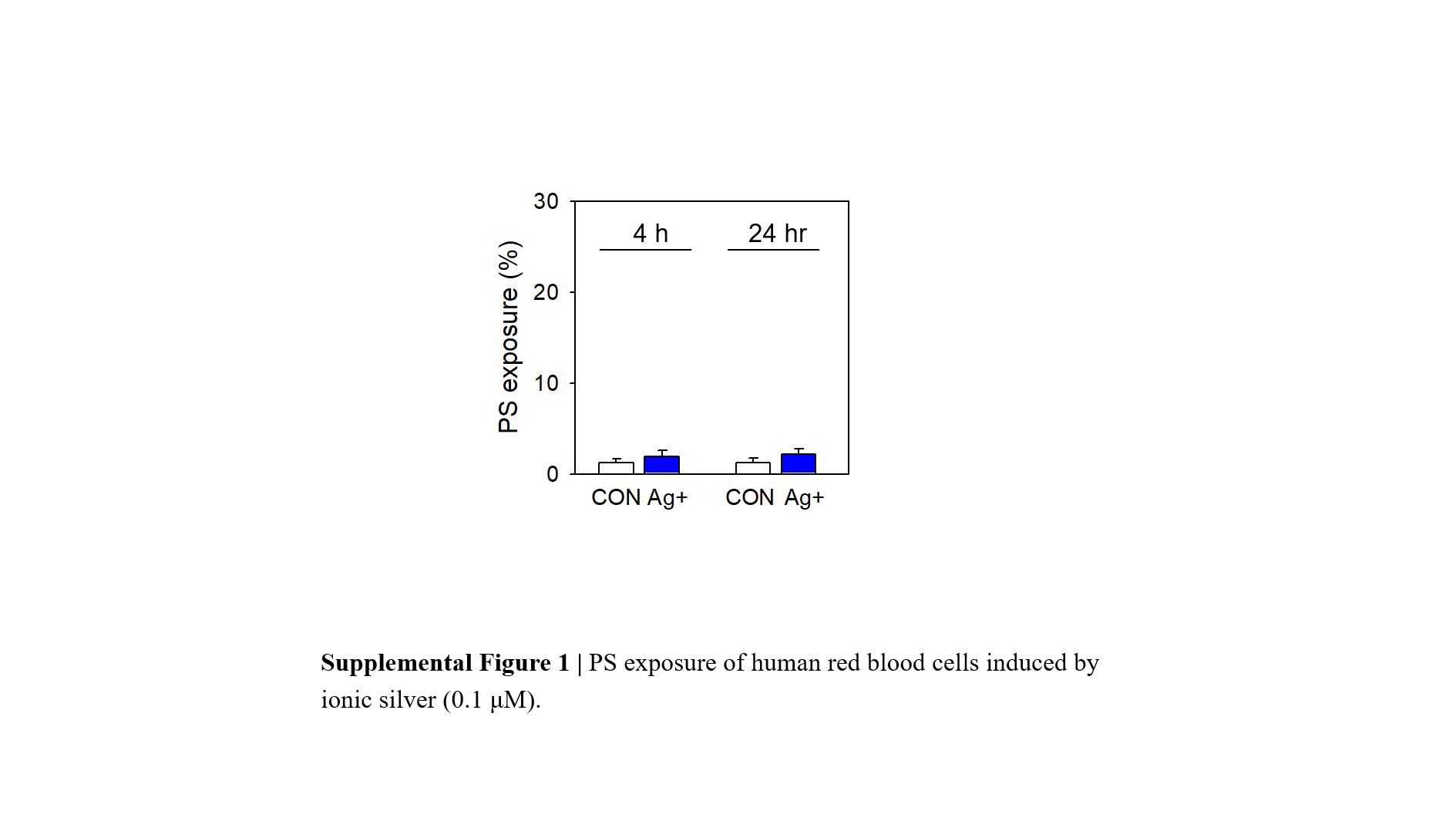

Supplement: Supplementary file 1 — Figure S1. PS exposure of human red blood cells induced by ionic silver (0.1 μM). (TIF 208 kb) [file 12989_2019_292_MOESM1_ESM.tif]

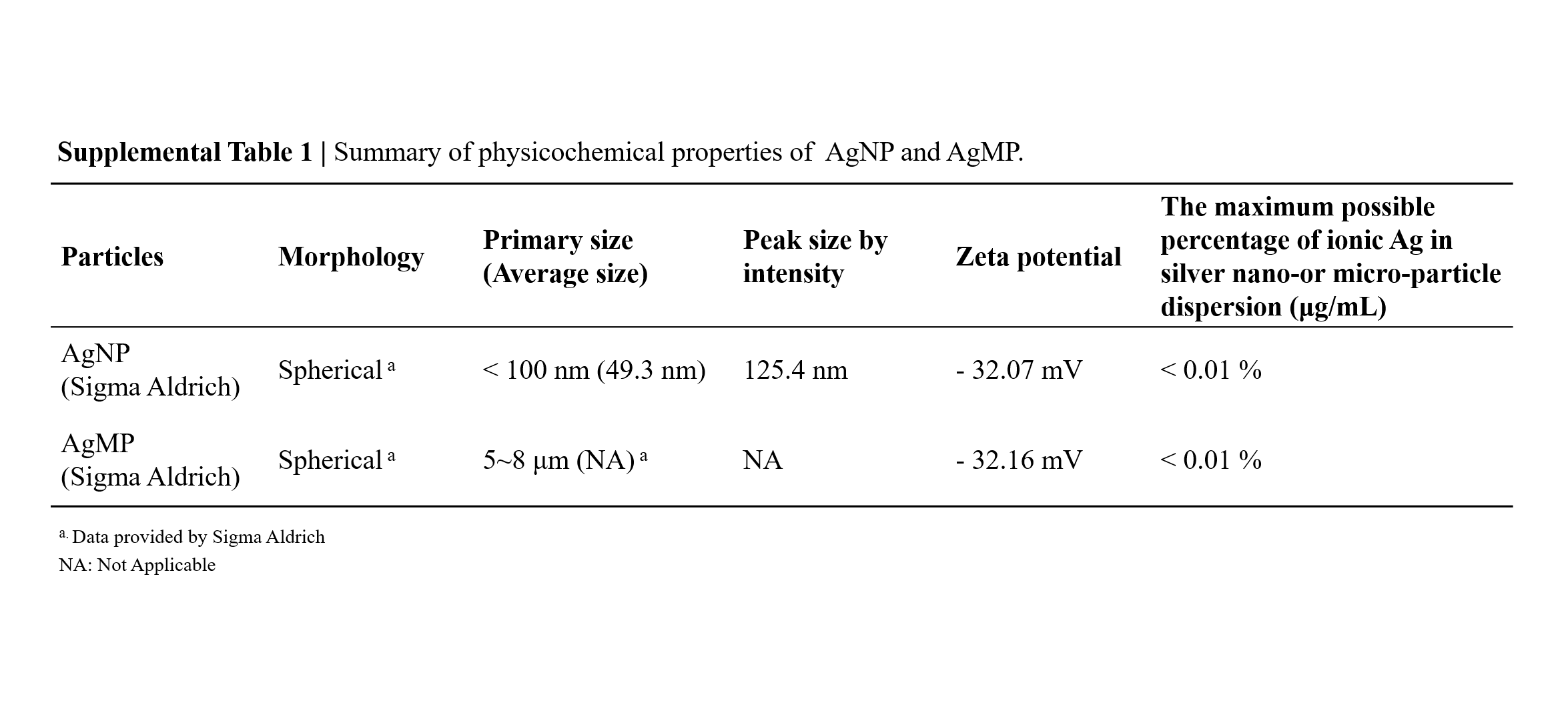

Supplement: Supplementary file 2 — Table S1. Summary of physicochemical properties of AgNP and AgMP. (TIF 314 kb) [file 12989_2019_292_MOESM2_ESM.tif]

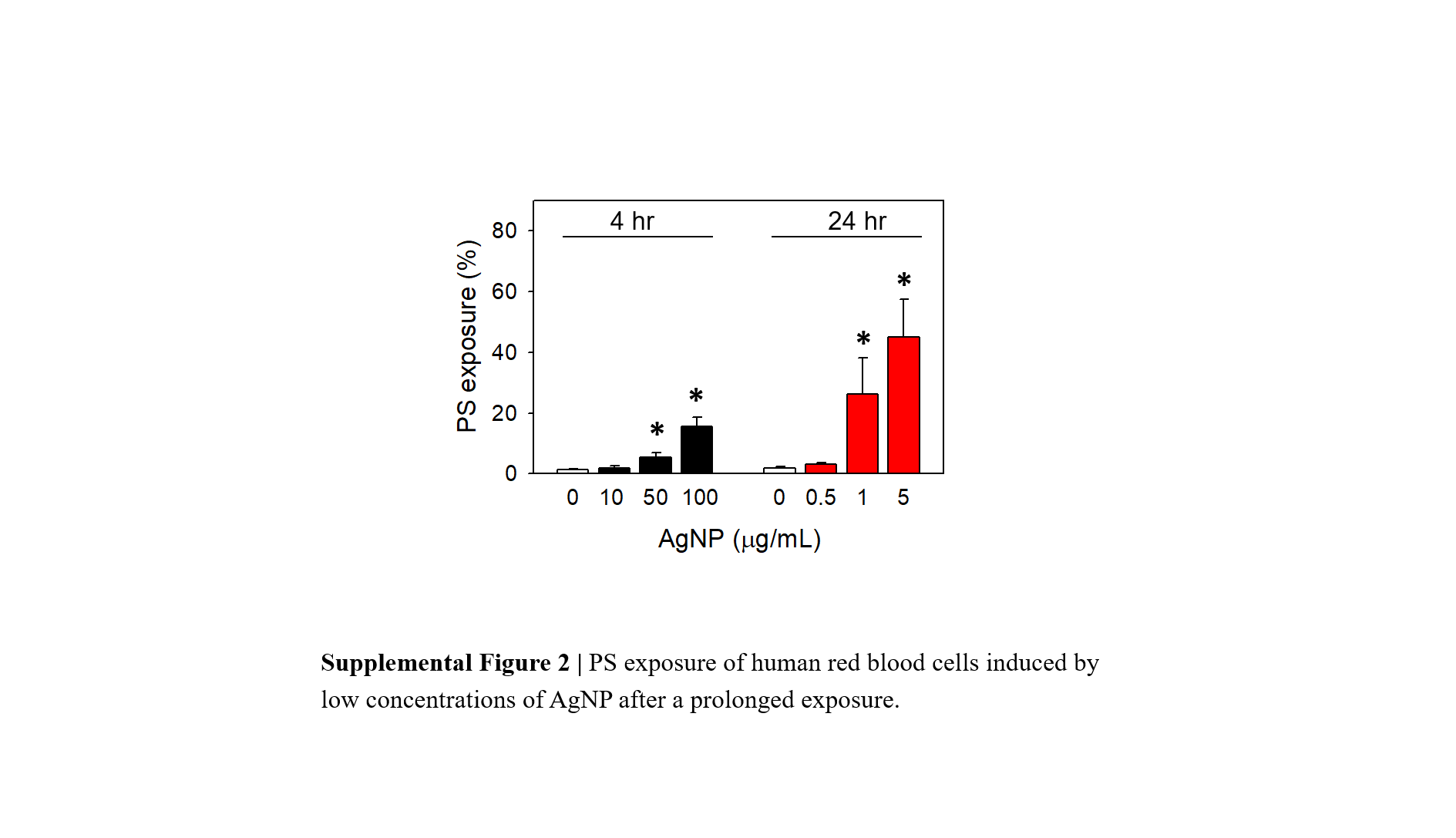

Supplement: Supplementary file 3 — Figure S2. PS exposure of human red blood cells induced by low concentrations of AgNP after a prolonged exposure. (TIF 232 kb) [file 12989_2019_292_MOESM3_ESM.tif]
